# The Role of Impaired Mitochondrial Dynamics in MFN2-Mediated Pathology

**DOI:** 10.3389/fcell.2022.858286

**Published:** 2022-03-24

**Authors:** Mashiat Zaman, Timothy E. Shutt

**Affiliations:** ^1^ Cumming School of Medicine, University of Calgary, Calgary, AB, Canada; ^2^ Department of Biochemistry and Molecular Biology, University of Calgary, Calgary, AB, Canada; ^3^ Alberta Children’s Hospital Research Institute (ACHRI), Calgary, AB, Canada; ^4^ Hotchkiss Brain Institute, University of Calgary, Calgary, AB, Canada; ^5^ Department of Medical Genetics, University of Calgary, Calgary, AB, Canada

**Keywords:** mitochondria, MFN2, CMT2A, mitochondrial endoplasmic reticulum contact sites, mitochondrial dynamics, mtDNA, mitophagy, lipid homeostasis

## Abstract

The Mitofusin 2 protein (MFN2), encoded by the *MFN2* gene, was first described for its role in mediating mitochondrial fusion. However, MFN2 is now recognized to play additional roles in mitochondrial autophagy (mitophagy), mitochondrial motility, lipid transfer, and as a tether to other organelles including the endoplasmic reticulum (ER) and lipid droplets. The tethering role of MFN2 is an important mediator of mitochondrial-ER contact sites (MERCs), which themselves have many important functions that regulate mitochondria, including calcium homeostasis and lipid metabolism. Exemplifying the importance of MFN2, pathogenic variants in *MFN2* are established to cause the peripheral neuropathy Charcot-Marie-Tooth Disease Subtype 2A (CMT2A). However, the mechanistic basis for disease is not clear. Moreover, additional pathogenic phenotypes such as lipomatosis, distal myopathy, optic atrophy, and hearing loss, can also sometimes be present in patients with CMT2A. Given these variable patient phenotypes, and the many cellular roles played by MFN2, the mechanistic underpinnings of the cellular impairments by which MFN2 dysfunction leads to disease are likely to be complex. Here, we will review what is known about the various functions of MFN2 that are impaired by pathogenic variants causing CMT2A, with a specific emphasis on the ties between MFN2 variants and MERCs.

## 1 Introduction

Mitochondria are dynamic organelles capable of division, fusion and movement within the cell, processes that are often grouped together under the term mitochondrial dynamics (Fenton et al., 2021; Yapa et al., 2021). These dynamic events determine mitochondrial shape and are important for mitochondrial function. For example, mitochondrial fusion allows content exchange (Franco et al., 2016), which can be an important step in quality control as it allows complementation. Additionally, mitochondrial fission is required to generate smaller mitochondria for transport, or degradation *via* mitochondrial autophagy (mitophagy). Meanwhile, both fusion and fission are important for regulation of the mitochondrial genome (mtDNA) (Sabouny and Shutt, 2021), which encodes thirteen proteins essential for oxidative phosphorylation, is present in hundreds of copies per cell, and is packaged into protein assemblies referred to as nucleoids, which are distributed throughout the mitochondrial network.

Expanding our understanding of the dynamic nature of mitochondria, we now recognize that mitochondria interact with many other organelles. These connections between the organelles are associated with multiple functions, including exchange of molecules, signaling and biochemical pathways. While many types of mitochondrial contacts have been documented [e.g. lysosomes (Peng et al., 2020), the nucleus (Eisenberg-Bord et al., 2021), lipid droplets (Kilwein and Welte, 2019), the plasma membrane (Meschede et al., 2020) and peroxisomes (Sargsyan and Thoms, 2020)], the most well-studied mitochondrial contact is with the endoplasmic reticulum (ER), which we will refer to here as mitochondrial-ER contacts (MERCs).

**FIGURE 1:**
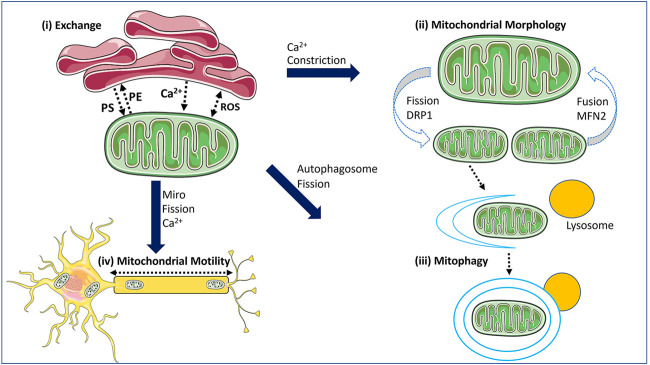
The functions of MERCs within the cell. Mitochondrial-ER contact sites (MERCs) mediate several processes. (i) MERCs mediate the exchange of signalling molecules such as calcium (Ca^2+^) and reactive oxygen species (ROS), as well as lipids. (ii) MERCs play a key role in regulating mitochondrial fission and fusion, as they can mark sites where these events occur with the ER wrapping around mitochondrial tubules to initiate constriction, and via calcium. (iii) MERCs have functional ties to mitophagy, with recruitment of mitophagy factors and formation of the autophagosome occurring at MERCs. Mitophagy is also linked to mitochondrial fission. (iv) MERCs impact mitochondrial motility, which requires fission to generate mitochondrial fragments for transport, and via the mitochondrial transport proteins MIRO1/2 which are linked to MERC functions and are regulated by calcium. The schematics used in the figures were taken from Smart Servier (2021).

Similar to how impairments in mitochondrial fission and fusion are now recognized to cause mitochondrial dysfunction and are linked to disease (Whitley et al., 2019; Chan, 2020), we are now beginning to appreciate the role of impaired MERCs in pathologies linked to mitochondrial dysfunction. In this review, we will examine what is known about how pathogenic variants in *MFN2*, which encodes a multifunctional mitochondrial protein, impact several dynamic mitochondrial processes, including mitochondrial fusion and MERCs. First, however, we will provide an overview of MERCs, their functions (Figure 1), and the roles of MFN2.

### 1.1 Mitochondrial—ER Contacts

MERCs were first observed by electron microscopy over 70 years ago (Bernhard and Rouiller, 1956; Copeland and Dalton, 1959). To date, electron microscopy remains a common method to study MERCs, although improved imaging technologies have refined our understanding of these sites, which can be distinct contacts between mitochondria and smooth or rough ER, at typical distances between 10 and 80 nm in length (Moltedo et al., 2019). Notably, MERCs can be further classified by their thickness (the gap between mitochondria and ER membranes), and their length, which are likely linked to their functionality (Giacomello and Pellegrini, 2016). Additionally, MERCs can also be estimated using a variety of fluorescence-based assays in both fixed and live cells (Giamogante et al., 2020). Some of these imaging approaches include standard immunofluorescent colocalization, proximity ligations assays (Benhammouda et al., 2021), and reporters such as split GFP reporters (Cieri et al., 2018; Yang et al., 2018) or resonance energy transfer systems such as FRET (Csordás et al., 2010) or BRET (Hertlein et al., 2020). Notably, these advances in live-cell imaging allow the study MERC dynamics, which are poorly understood. Meanwhile, standard assays for Ca^2+^ and lipid flux can also provide insight into the functionality of MERCs (Larrea et al., 2019), while cell fractionation methods can be used to isolate MERC fractions (Wieckowski et al., 2009). In this regard, another term that is frequently connected to contacts between mitochondria and the ER is mitochondria-associated ER membranes (MAMs), which are defined as a biochemical fraction first identified in the 1990s (Vance, 1990). Though the two terms are often used interchangeably in papers, MAMs are distinct from MERCs based on their technical definitions (Giacomello and Pellegrini, 2016).

**FIGURE 2:**
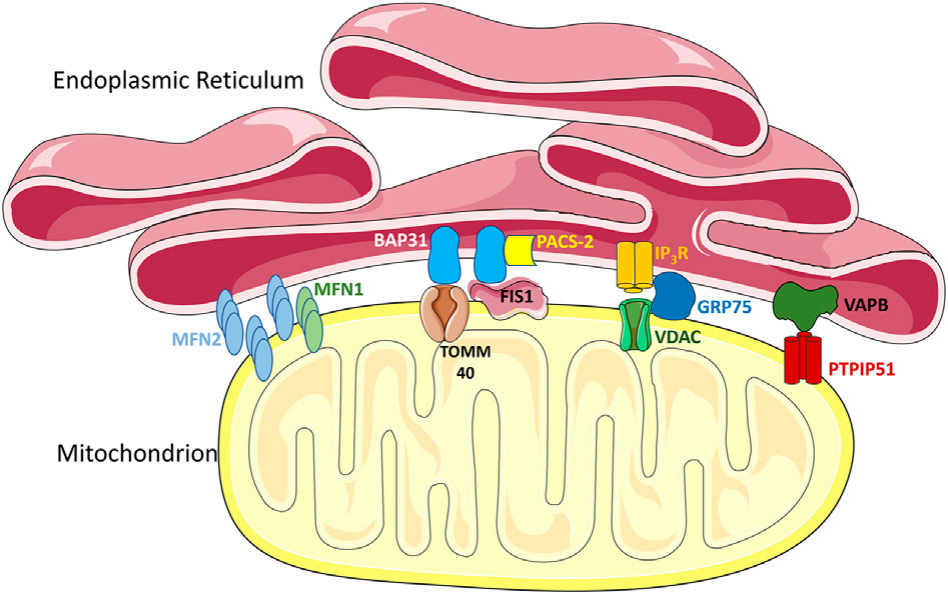
MERC tethers. Multiple proteins in the ER and the outer mitochondrial membrane (OMM) act as tethering complexes that can stabilize MERCs. Notably, MFN2 can localize on both the mitochondrial outer membrane and the ER membrane, allowing MFN2 on the ER membrane to form homotypic contacts with MFN2 or heterotypic contacts with MFN1 on the OMM. BAP-31 on the ER membrane can bind to TOMM40 and FIS1 in the OMM, interactions which are mediated by PACS-2. Interactions between IP_3_R in the ER and VDACs in the OMM, are mediated *via* GRP75, and are important in mediating Ca^2+^ transfer. Another interaction shown is between VAPB in the ER and PTPIP51 in the mitochondria, which forms a key tethering complex regulating the spatial conformation of MERCs. The schematics used in the figures were taken from Smart Servier (2021).

While contact sites between mitochondria and the ER are conserved throughout evolution, their composition varies across species. In yeast, MERCs are mediated by the ER-mitochondria encounter structures (ERMES), which was first identified through a genetic screen (Kornmann et al., 2009). Interactions between the protein subunits of the ERMES facilitates the formation of contacts and plays a key role in terms of function. However, mammalian cells do not encode any of the subunits seen in the ERMES. Instead, MERCs in mammalian cells are mediated by a collection of different protein tethers with structural and/or functional relevance (Giacomello and Pellegrini, 2016) (Figure 2). The reasons for this increased complexity in mammalian MERCs are unknown, but could reflect the specialization of MERCs for the many different functions that they perform (Salvador-Gallego et al., 2017). Notably, the MFN2 protein, the focus of this review, was one of the first mammalian MERC tethers identified (de Brito and Scorrano, 2008a).

### 1.2 Mitochondrial—ER Contact Functions

MERCs are crucial to many biological processes. For instance, MERCs are required to mediate the transfer of metabolites between mitochondria and ER, and they facilitate communication between these organelles, as they are involved in the transfer of signalling molecules such as calcium (Ca^2+^) and reactive oxygen species (ROS) (Booth et al., 2021). MERCs can also play a key regulatory role in mitochondrial dynamics, including maintenance of the mitochondrial network and mitophagy. Apart from these roles, MERCs are also connected to general autophagy (Yang et al., 2018), insulin signalling (Giorgi et al., 2010), nutrient sensing (Theurey et al., 2016), and the unfolded protein response (Mori et al., 2013). Here we focus on the roles of MERCs that are of relevance to MFN2 [Figure 1].

#### 1.2.1 Calcium Homeostasis

Ca^2+^, an important intracellular signalling molecule, is involved in an array of cellular processes such as cell migration, apoptosis, initiation of transcription, neurotransmission, and exocytosis, to name a few (Berridge et al., 2003). The ER is a key storage repository of intracellular Ca^2+^, and Ca^2+^ that is released from the ER can be taken up by mitochondria. However, the proximity between mitochondria and ER, and thus MERCs, are critical for this process (Rizzuto et al., 1998). As the importance of MERCs for Ca^2+^ has been reviewed extensively elsewhere (Krols et al., 2016; Lim et al., 2021; Loncke et al., 2021; Vallese et al., 2020), here we provide only a brief overview of the key players. First, Ca^2+^ is released from the ER *via* IP3R channel. Next, Ca^2+^ passes across the outer mitochondrial membrane (OMM) through the voltage-gated anion channels (VDACs), while the mitochondrial calcium uniporter (MCU) transports Ca^2+^ across the inner mitochondrial membrane (IMM). Notably, VDAC1 and IP3R act in concert as MERC tethering proteins (de Stefani et al., 2012) (Figure 2), an interaction that is facilitated by the GRP75 chaperone protein (Szabadkai et al., 2006). Following import into mitochondria, Ca^2+^ has physiological consequences, as key mitochondrial processes are mediated by Ca^2+^ in the matrix. For example, Ca^2+^ has a regulatory role in the Krebs cycle, through positive control of mitochondrial dehydrogenases (Gutiérrez et al., 2020). Meanwhile, Ca^2+^ signalling levels can also control ATP production in an influx dependent manner (Luongo et al., 2015). Thus, MERCs play a key role in mediating an intricate equilibrium in Ca^2+^ signalling that can mediate mitochondrial function.

#### 1.2.2 Lipid Metabolism

Lipids are important molecules that help make up membranes, and which can also be catabolised to produce energy. With respect to lipid synthesis, the enzymes that generate different lipid species can be present in various compartments, including the ER and mitochondria. As such, the transfer of lipids between these organelles is important, and is a process that was first shown to occur *via* MAMs in the 1990s (Vance, 1990). A crucial example of this process involves two key membrane phospholipids, phosphatidylserine (PS) and phosphatidylethanolamine (PE). PS, which is synthesized in the ER, is the precursor for PE that is produced by the mitochondrial decarboxylase PISD. The importance of this pathway is highlighted by the fact that *PISD* knockout is embryonic lethal in mice (Vance and Tasseva, 2013), and the recent discovery of pathogenic variants in *PISD* (Girisha et al., 2019; Peter et al., 2019; Zhao et al., 2019). Critically, given the hydrophobic nature of lipids, the transfer of PS from the ER to mitochondria requires MERCs (Vance and Tasseva, 2013), while MFN2 has been directly implicated in transferring PS from the ER to mitochondria (Hernández-Alvarez et al., 2019). Similarly, steroidogenesis is a process that is impacted by MERCs, as cholesterol synthesized in the ER needs to be imported into mitochondria. Once in the mitochondria, cholesterol is converted to pregnenolone, the precursor for all steroid hormones, many of which are produced by ER-localized enzymes (Klecker et al., 2014; Booth et al., 2016). Thus, MERCs play a critical role in lipid metabolism by facilitating the transport of lipids back and forth between mitochondria and the ER.

#### 1.2.3 Mitochondrial Fission

The first step in mitochondrial fission is a constriction event of the mitochondrial tubule that allows the mitochondrial fission protein, dynamin-related-protein 1 (Drp1), to be recruited to the OMM by a variety of different adaptor proteins (e.g., MFF, MID49, MID51, FIS1). DRP1 then oligomerizes around mitochondrial tubules and further constricts to drive mitochondrial fission. Notably, mitochondrial contacts sites with the ER (Friedman et al., 2011) or lysosomes (Wong et al., 2018) can both initiate mitochondrial fission events. With respect to MERCs, the ER wraps around mitochondria to provide the constriction event that initiates fission. This constriction is mediated by the actin-myosin cytoskeleton (Hatch et al., 2014; Korobova et al., 2014), and is regulated by INF2 on the ER (Korobova et al., 2013) and SPIRE1C in the OMM (Manor et al., 2015) In addition, actin polymerization during this initial constriction increases MERCs to promote uptake of Ca^2+^ from the ER to the mitochondrial matrix, which then activates constriction of the IMM (Chakrabarti et al., 2018). As such, MERCs can play a pivotal role in mediating mitochondrial fission. Notably, MERCs are also reported to mark sites of mtDNA replication preceding fission events (Lewis et al., 2016). Critically, not all fission events are created equal, as fission events that occur in the middle of mitochondria and are mediated by ER constrictions and MFF, seem to be linked to mitochondrial biogenesis and replication of the mtDNA (Kleele et al., 2021). Meanwhile, fission events that occur near the ends of mitochondrial tubules, and are initiated by lysosome contacts, seem to be fated for mitophagy (Kleele et al., 2021). Given the critical role of MERCs in fission, it seems likely that alterations in MERCs will impact fission dynamics. In this regard, either too much or too little mitochondrial fission can prove to be detrimental.

#### 1.2.4 Mitochondrial Fusion

Mitochondrial fusion is also a multi-step process. Fusion is initiated by a tethering process, which is mediated by the Mitofusin proteins MFN1 and MFN2 in the OMM (Chen et al., 2003). Once this connection has been made, the OMM can fuse. Next, fusion of the inner mitochondrial membrane (IMM) is mediated by OPA1 (Griparic et al., 2004; Liu et al., 2009). Notably, there is evidence for a role of MERCs in helping to mediate mitochondrial fusion, as MERCs also appear to demarcate sites of fusion (Abrisch et al., 2020), although the underlying mechanism remains largely unknown. In this sense, MERCs are proposed to mediate fission and fusion cycles that have been reported previously (Liu et al., 2009). Thus, through roles in both fission and fusion, MERCs ultimately help determine the shape and health of the mitochondrial network.

#### 1.2.5 Mitophagy

In addition to mitochondrial fission and fusion, MERCs are also implicated in another dynamic process, mitophagy, which mediates the systematic removal of damaged or dysfunctional mitochondria. Although mitochondrial fission is required for mitophagy, as hyperfused mitochondrial networks are too big to be degraded (Gomes et al., 2011; Rambold et al., 2011), as noted above, mitochondria destined for mitophagy do not appear to divide at sites that are mediated by MERCs under steady-state conditions (Kleele et al., 2021). However, it is possible that MERC-mediated mitochondrial fission is linked to mitophagy under certain conditions. Nonetheless, MERCs are involved in mitophagy in other ways. Work in yeast provided some of the initial insight into the connection between mitophagy and MERCs, as a direct interaction between the ERMES subunit Mmm1 and the autophagy protein Atg8 has been observed, along with the absence of Mmm1 leading to malformed mitophagosomes (Böckler and Westermann, 2014). Meanwhile, in mammalian cells, the autophagy protein ATG14 accumulates in MAM fractions, suggesting that MERCs are potential sites of autophagosome initiation required for mitophagy (Hamasaki et al., 2013). Further supporting this notion, the mitophagy proteins PINK1 and BECN1 also accumulate at MAMs and promote ER tethering (Gelmetti et al., 2017), while disruption of MERCs impairs mitophagosome formation and mitophagy (Moulis et al., 2019). Although there is more work to be done to understand the connections between MERCs and mitophagy, it is clear that MERCs are important in regulating this aspect of mitochondrial quality control.

### 1.3 MFN2

**FIGURE 3:**
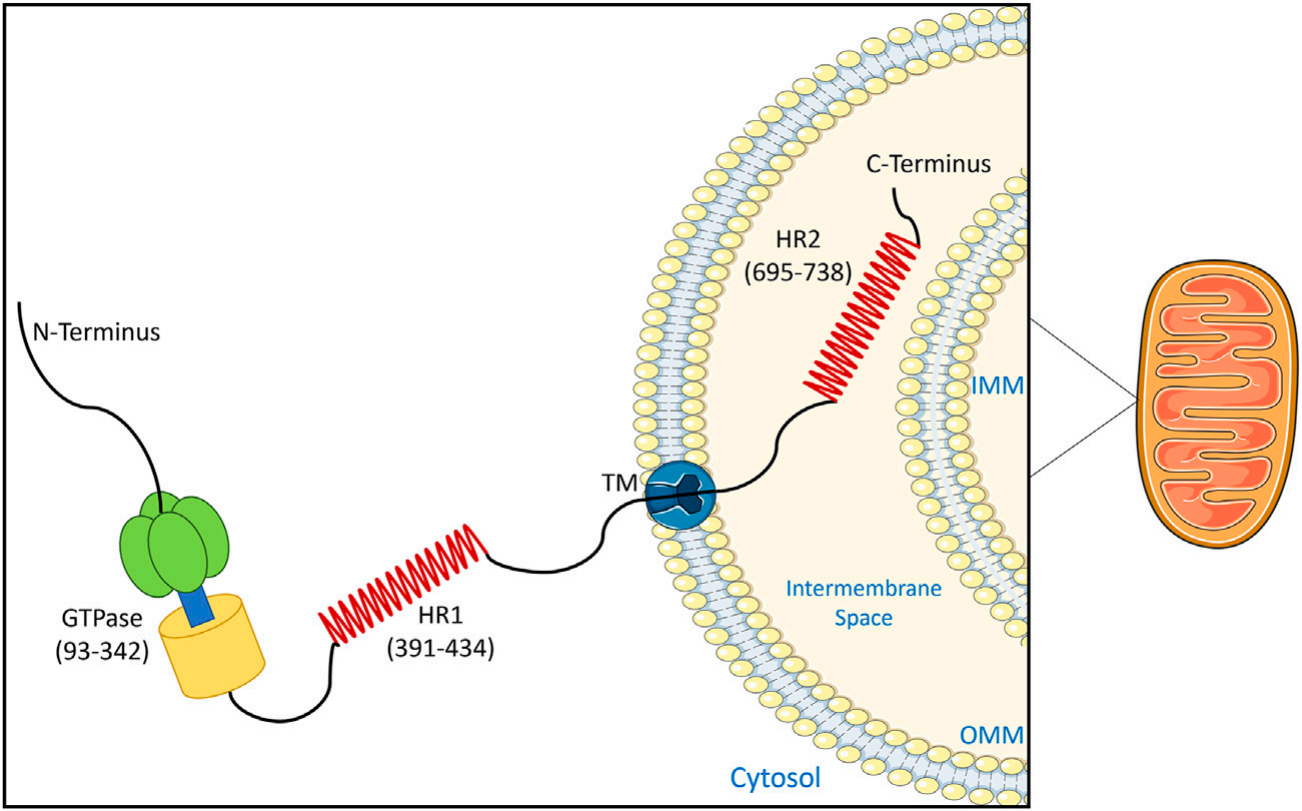
Topology of MFN2. This schematic shows the domain arrangement and spatial structure of the MFN2 protein on the mitochondrial outer membrane. The 757 amino acid protein contains a GTPase domain and heptad repeat domain (HR1) exposed to the cytosol, and a second heptad repeat domain (HR2) that localizes to the mitochondrial intermembrane space. The schematics used in the figures were taken from Servier Medical Art by Servier.

Humans encode two Mitofusin isoforms MFN1 and MFN2, which were initially defined for their essential role in mediating fusion of the outer mitochondrial membrane (Chen et al., 2003). MFN1 and MFN2 are conserved homologs, which can mediate mitochondrial fusion in either a homotypic (MFN1/MFN1 or MFN2/MFN2) or heterotypic fashion (MFN1/MFN2) (Hoppins et al., 2007). These integral OMM proteins comprise an N-terminal domain exposed to the cytosol, which contains a GTPase domain, and a coiled-coiled heptad repeat domain (HR1). Meanwhile, the C-terminal domain, which is separated by a transmembrane domain, contains a second heptad repeat domain (HR2) that localizes to the inter-membrane space (Figure 3) (Mattie et al., 2018). However, despite these similarities, MFN1 and MFN2 are not equivalent, and are not entirely redundant. For example, MFN1 has higher GTPase activity and tethering capabilities (Ishihara et al., 2004). Meanwhile, MFN2 is distinct in that it can also localize to the ER, and has additional cellular roles, one of which is the tethering MERCs (de Brito and Scorrano, 2008b).

MFN2 in the ER membrane can form either homotypic or heterotypic contacts with either MFN1 or MFN2 in the OMM (de Brito and Scorrano, 2008a; Casellas-Díaz et al., 2021), and initial studies showed that MFN2 depletion leads to reduced numbers of MERCs (de Brito and Scorrano, 2008b). Moreover, functional studies showed that MFN2 is important for phospholipid transfer (Area-Gomez et al., 2012) and Ca^2+^ uptake (de Brito and Scorrano, 2008b), consistent with a role in mediating MERCs. However, there is some recent debate on the role of MFN2 in mediating MERCs, as some groups have observed that loss of MFN2 leads to increased MERCs, resulting in the suggestion that MFN2 acts as a spacer, rather than a tether (Cieri et al., 2018). In response, counterevidence for the role that MFN2 plays as a tether MERCs has been provided both in cultured cells (Naon et al., 2016), and *in vivo* (Han et al., 2021). In particular, this later *in vivo* work in the brain is relevant to the disease roles of MFN2. Critically, conditional MFN2 knockout reduced MERCs, while a reciprocal MFN2 overexpression increased MERCs. Additional support for the role of MFN2 in MERC formation was evidenced by the fact that modulating MFN2 expression also impacted the interactions between other MERC tethers (e.g., IP3R3-VDAC1 and VAPB-PTPIP51) (Han et al., 2021). Though the reasons for the discrepancies between these various groups is unknown, it is clear nonetheless that MFN2 is involved in mediating MERCs.

In addition to its roles in mediating mitochondrial fusion and MERCs, which both play key roles in mediating mitochondrial function, MFN2 has several additional intracellular roles. One of these roles is in mitophagy, as MFN2 acts as a receptor for PINK1-Parkin mediated mitophagy (Chen and Dorn, 2013). Meanwhile, MFN2 is also involved in mitochondrial transport (Baloh et al., 2007) via direct interactions with the mitochondrial transport machinery (Misko et al., 2010), including the MIRO1/2 transport proteins, which notably also have functional ties to MERCs (Modi et al., 2019). Finally, MFN2 is also implicated in lipid metabolism, as it can tether mitochondria to lipid droplets (Boutant et al., 2017), and is also reported to participate directly in the transfer of phosphatidylserine from the ER to mitochondria (Hernández-Alvarez et al., 2019).

The functions performed by MFN2 can also have downstream consequences on mitochondrial function. For example, fusion regulates mitochondrial ultrastructure, which can impact oxidative phosphorylation by altering cristae organization and supercomplex formation (Baker et al., 2019; Glancy et al., 2020) Meanwhile, MERCs can influence oxidative phosphorylation via Ca^2+^ (Gutiérrez and Simmen, 2018). Additionally, MFN2 can impact the stability of mtDNA, which encodes essential proteins required for oxidative phosphorylation. Both depletion of mtDNA copy number, and increased incidence of mtDNA deletions are associated with MFN2 dysfunction (Rouzier et al., 2012; Vielhaber et al., 2013), likely via its fusion role, which is important for the proper distribution of proteins that regulate mtDNA (Ramos et al., 2019). Together, these various MFN2 functions begin to explain the reduced mitochondrial respiration observed in cells lacking MFN2 (Kawalec et al., 2015).

Given these pivotal cellular functions, it should come as no surprise that MFN2 is key to organismal development, as complete loss is lethal in mice (Chen and Chan, 2010), and larval lethal in *Drosophila melanogaster* (Sandoval et al., 2014). Moreover, MFN2 dysfunction can lead to disease as mutations in MFN2 cause the peripheral neuropathy Charcot-Marie-Tooth Disease Type 2A (CMT2A). In this regard, another difference between MFN1 and MFN2, is the fact that pathogenic variants have only been described in MFN2, not MFN1. The reason for this discrepancy was hypothesized to be due to the low level of MFN1 expression in neurons, where the effects of MFN2 dysfunction manifest, such that MFN1 cannot compensate for loss of MFN2 function (Detmer and Chan, 2007), and this idea is supported by data showing that MFN2 is more highly expressed relative to MFN1 in mouse dorsal root ganglia (Kawalec et al., 2014). However, as we shall discuss later, the pathology caused by pathogenic MFN2 variants may also reflect the additional roles performed by MFN2, on top of mitochondrial fusion.

### 1.4 MFN2 and Charcot-Marie-Tooth Disease Type 2A

CMT is a peripheral neuropathy that is typically classified based on neurophysiological features as being either demyelinating, with reduced conduction velocity, or axonal, with reduced amplitudes. However, intermediate forms of CMT exhibiting both features do exist (Berciano et al., 2017). While the first descriptions of CMT are from the 1880s, when it was characterized as muscular atrophy (Dorn, 2020), there are now multiple subtypes of CMT recognized based on their clinical presentation and genetic causes. Of these subtypes, CMT2A is part of the axonal category (Feely et al., 2011), and is characterized by weakness, numbness, and pain, particularly in distal regions of the body, including the limbs. The typical symptoms of CMT2A and related prognosis show degeneration in motor activity and general worsening of the patient condition, making the scenario a progressively deteriorating one.

Pathogenic variants in *MFN2* (O’Toole et al., 2008; Chang et al., 2011; Saxton and Hollenbeck, 2012) are the leading cause of CMT2A, with over one hundred described to date (Züchner et al., 2004). Though most *MFN2* variants linked to CMT2A are autosomal dominant, recessive homozygous or compound heterozygous variants have also been described in patients with more severe disease (Zhang et al., 2017). However, many of these MFN2 variants have not been studied functionally, and it remains unknown exactly how MFN2 dysfunction leads to CMT2A.

Impairments in mitochondrial dynamics are often linked to peripheral neuropathies (Sharma et al., 2021a). One way through which impaired mitochondrial dynamics are thought to contribute to peripheral neuropathy is via reduced mitochondrial motility (Schiavon et al., 2021), which can occur bi-directionally along axons (O’Toole et al., 2008; Saxton and Hollenbeck, 2012). Mitochondrial transport is obviously important in the long axons found in neurons, as synapses present in the distal region of neurons require mitochondria to provide the energy. However, other types of cellular dysfunction that are also linked to MFN2 dysfunction, such as altered lipid droplets and impaired MERCs, can also cause peripheral neuropathy (Sharma et al., 2021a). Thus, it is not clear that the peripheral neuropathy in CMT2A is due exclusively to impaired mitochondrial motility.

In addition to peripheral neuropathy, patients with MFN2 variants can sometimes also present with additional phenotypes, including optical atrophy (Leonardi et al., 2015), sensorineural hearing loss (Chung et al., 2006), cerebellar ataxia (Sharma et al., 2021b) and multiple systemic lipomatosis (Capel et al., 2018), to name a few. The reasons for this phenotypic variability due to different variants in the same gene remain unknown. While it is tempting to speculate that different pathogenic variants have distinct impacts on the various functions performed by MFN2, this notion remains untested. In this review, we will examine what is known about how different MFN2 variants impact different MFN2 functions, with a specific focus on MERCs.

## 2 Overview of MFN2 Functions Impacted by MFN2 Variants

Only a relatively small subset of the over 100 pathogenic MFN2 variants linked to CMT2A have been studied for their effects on MFN2 functions. Moreover, a comprehensive picture is lacking, as separate groups have examined different MFN2 variants for various MFN2 functions at distinct times in different cells using different approaches. Here, we provide an overview of the patchwork understanding of how different pathogenic MFN2 variants impact the roles of MFN2 and mitochondrial functions (Tables 1, 2).

**TABLE 1:** Pathogenic MFN2 variants functionally characterized for their effects on mitochondrial morphology or MERCs. A list of pathogenic MFN2 variants, defined by their amino acid changes, that have been studied for their effects on mitochondrial morphology or MERCs. Associated patient phenotypes are indicated, and a description of how mitochondrial morphology and MERCs are altered is provided. Unless otherwise specified, the cell type or tissue investigated are indicated as follows: Patient derived fibroblasts [FIB]; mouse embryonic fibroblasts with *Mfn1/Mfn2* double KO and variant re-expressed [MEF]; myelinated and non-myelinated nerve [NER]; skin and muscle biopsy samples [SKM]. re-expression in rat neurons [NEU]. Relevant references are cited.

MFN2 variant	Domain location	Phenotype	Inheritance	Mitochondrial morphology	Effect on MERCs	References
M21V	N-terminus	Early onset-CMT2A	Autosomal Dominant	Normal [FIB]	—	Loiseau et al. (2007)
V69F	N-terminus	CMT2A	Autosomal Dominant	Aggregated [NEU]	—	(Baloh et al., 2007; Detmer and Chan, 2007)
				Normal [FIB]	—	
L76P	N-terminus	Early onset-CMT2A	Autosomal Dominant	Aggregation [MEF]/[NEU]	—	(Verhoeven et al., 2006; Baloh et al., 2007; Detmer and Chan, 2007)
G80V	N-terminus	CMT2A	Autosomal Dominant	Hyperfusion [FIB]	—	Codron et al. (2016)
R94Q	GTPase	Early onset-CMT2A	Autosomal Dominant	Fragmented [MEF] *Mfn2* ^−/−^Aggregated [NEU]	Reduced Number and Length [Neurons and FIB]	(Baloh et al., 2007; Neusch et al., 2007; Misko et al., 2010; Bernard-Marissal et al., 2019; Wolf et al., 2019)
R94W	GTPase	Early onset-CMT2A	Autosomal Dominant	Fragmented [MEF-knock in]	—	(Züchner et al., 2006; Strickland et al., 2014)
R95G	GTPase	Early onset-CMT2A	Autosomal Dominant	—	—	Dankwa et al. (2019)
K98E	GTPase	Early onset-CMT2A	Autosomal Dominant	Round	—	Vallat et al. (2008)
				Small		
				Aggregated [NER]		
V99M	GTPase	CMT2A	Autosomal Dominant	—	—	Ma et al. (2021)
R104Q	GTPase	CMT2A	Autosomal Dominant	Hyperfused [FIB]	—	Codron et al. (2016)
R104W	GTPase	Early onset-CMT2A	Autosomal Dominant	Round	—	(Vallat et al., 2008; Calvo et al., 2009)
				Small		
				Aggregated [NER]		
T105M	GTPase	Early onset-CMT2A	Not Specified	Normal [FIB]	—	(Lawson et al., 2005; Amiott et al., 2008)
G127D	GTPase	CMT2A	Autosomal Dominant	—	—	(Chung et al., 2006; Beręsewicz et al., 2018)
G127V	GTPase	Late onset-CMT2A	Autosomal Dominant	—	—	(Engelfried et al., 2006; Beręsewicz et al., 2018)
A164V	GTPase	CMT2A	Autosomal Dominant	Round	—	Vallat et al. (2008)
				Small		
				Aggregated [NER]		
H165R	GTPase	CMT2A	Autosomal Dominant	—	—	(Chung et al., 2006; Beręsewicz et al., 2018)
A166T	GTPase	Early onset-CMT2A	Autosomal Dominant	Normal [FIB]	—	Loiseau et al. (2007)
G176S	GTPase	Early onset-CMT2A	Autosomal Recessive	—	—	(Gonzaga-Jauregui et al., 2015; Iapadre et al., 2018)
D210V	GTPase	Early onset-CMT2A Optic Atrophy	Autosomal Dominant	Fragmented [FIB]	—	Rouzier et al. (2012)
D210Y	GTPase	Early onset-CMT2A	Autosomal Dominant	Abnormal cristae and reduced size [SKM]	—	Renaldo et al. (2012)
I213T	GTPase	Early onset-CMT2A	Autosomal Dominant	Normal [FIB]	—	(Lawson et al., 2005; Amiott et al., 2008; Yang et al., 2021)
D214N	GTPase	Early onset-CMT2A	Autosomal Dominant	Round	—	Vallat et al. (2008)
				Small		
				Aggregated [NER]		
F216S	GTPase	Early onset-CMT2A	Autosomal Recessive	Round	—	Vallat et al. (2008)
				Small		
				Aggregated [NER]		
F240I	GTPase	CMT2A	Not Specified	Normal [FIB]	—	Amiott et al. (2008)
V244L	GTPase	Early onset-CMT2A	Autosomal Dominant	—	—	Yang and Li, (2016)
R250Q	GTPase	CMT2A	Autosomal Dominant	—	—	McCorquodale et al. (2011)
P251A	GTPase	Early onset-CMT2A Tremoring	Autosomal Dominant	Fragmented [MEF] Aggregated [NEU]	Reduced [MEF]	(Züchner et al., 2004; Baloh et al., 2007; Detmer and Chan, 2007; Basso et al., 2018)
R259C	GTPase	CMT2A	Autosomal Dominant	—	—	Leonardi et al. (2015)
R259L	GTPase	CMT2A	Autosomal Dominant	—	—	Ajroud-Driss et al. (2009)
V273G	GTPase	Early onset-CMT2A	Autosomal Dominant	Normal [FIB]	—	(Lawson et al., 2005; Amiott et al., 2008)
R274W	GTPase	CMT2A	Autosomal Dominant	Normal in Glucose Media	Enlarged ER morphology [FIB]	Beresewicz et al. (2017)
				Fragmented in Glucose-free media [FIB]		
R280H	GTPase	Early onset-CMT2A	Autosomal Dominant	Aggregated [NEU]	—	(Verhoeven et al., 2006; Baloh et al., 2007)
T362M	GTPase	CMT2A	Autosomal Dominant	Round	—	Vallat et al. (2008)
				Small		
				Aggregated [NER]		
R364Q	GTPase	CMT2A	Autosomal Dominant	Normal [FIB]	—	Loiseau et al. (2007)
R364W	Inter-Domain Space	Early onset-CMT2A	Autosomal Dominant	Normal [FIB]	Increased distance [FIB]	(Feely et al., 2011; Larrea et al., 2019)
M376V	Inter-domain space	Late onset-CMT2A	Autosomal Dominant	Normal [FIB]	Reduced distance [FIB]	(Xie et al., 2016; Larrea et al., 2019)
S378P	Inter-domain space	CMT2A	Not Specified	Normal [MEF] *Mfn2* ^−/−^	—	Samanas et al. (2020)
A383V	Inter-domain space	CMT2A	Autosomal Dominant	Increase in perinuclear mitochondria [FIB]	—	(Muglia et al., 2007; Rizzo et al., 2016; Samanas et al., 2020)
				Hyperfusion [MEF] *Mfn2* ^−/−^		
Q386P	Inter-domain space	Early onset-CMT2A	Autosomal Dominant	Normal [MEF] *Mfn2* ^−/−^	—	(Verhoeven et al., 2006; Samanas et al., 2020)
C390F	Inter-domain space	Early onset-CMT2A	Autosomal Dominant	Normal [MEF] *Mfn2* ^−/−^	—	Samanas et al. (2020)
C390R	Inter-domain space	Early onset-CMT2A	Autosomal Dominant	Round	—	Vallat et al. (2008)
				Small		
				Aggregated [NER]		
D414V	HR1	Early onset-optic atrophy, ataxia, sensorineural hearing loss	Autosomal Dominant	Fragmented [FIB]	Reduced number and size of contacts [FIB]	Sharma et al. (2021b)
R707W	HR2	Early onset-CMT2A	Autosomal Dominant	—	—	Nicholson et al. (2008)
R707W	HR2	Late onset-Lipomatosis	Autosomal Recessive	Fragmented [Lentiviral transfection of MFN2 knockdown U2OS cells]	—	(Sawyer et al., 2015; Rocha et al., 2017)
L734V	HR2	CMT2A	Not Specified	Normal [FIB]	—	Amiott et al. (2008)
W740S	C-terminus	Late onset-CMT2A	Autosomal Dominant	Normal [FIB]	Reduced in number and increased distance [FIB]	(Baloh et al., 2007; Larrea et al., 2019)
Q751X	C-terminus	Early onset-CMT2A	Autosomal Dominant	—	—	Verhoeven et al. (2006)

**TABLE 2:** Pathogenic MFN2 variants with additional cellular alterations. In addition to alterations in mitochondrial morphology and MERCs (Table 1), several other cellular phenotypes have been investigated for some MFN2 variants (e.g., mitochondrial respiration, mtDNA, and lipid droplets). The MFN2 variants and patient phenotypes described for these cellular phenotypes are indicated. Examples with no reported changes in cellular phenotypes are also included. Unless otherwise specified, the cell type or tissue investigated is indicated as follows: patient derived fibroblasts [FIB]; mouse embryonic fibroblasts with *Mfn1/Mfn2* double KO and variant re-expressed [MEF]; skeletal muscle samples [MUS]; re-expression in rat neurons [NEU]. Relevant references are cited.

Cellular phenotype	MFN2 variant(s)	Patient pathology	References
Decreased Mitochondrial Respiration	M21V [FIB]	CMT2A	(Loiseau et al., 2007; Vielhaber et al., 2013; Strickland et al., 2014; Sharma et al., 2021b)
	R94W [Mouse Tissue]	CMT2A	
	A166T [FIB]	CMT2A	
	V226_S229DEL [FIB]	CMT2A	
	R364Q [FIB]	CMT2A	
	M376V [FIB]	CMT2A	
	D414V [FIB]	Ataxia, sensorineural hearing loss and optic atrophy	
Normal Mitochondrial Respiration	V69F [NEU]	CMT2A	(Baloh et al., 2007; Amiott et al., 2008)
	L78P [NEU]	CMT2A	
	R94Q [NEU]	CMT2A	
	T105M [FIB]		
	I213T [FIB]		
	P251A [NEU]		
	V273G [FIB]		
	R280H [NEU]		
	W740S [NEU]		
Presence of mtDNA deletions	Q74R [FIB]	CMT2A	(Rouzier et al., 2012; Vielhaber et al., 2013)
	D210V [FIB]	CMT2A	
	V226_S229del [FIB]/[MUS]		
	M376V [FIB]/[MUS]		
	R707P [FIB]/[MUS]		
Decreased mtDNA copy number	Q74R [FIB]	CMT2A	(Renaldo et al., 2012; Vielhaber et al., 2013; Beresewicz et al., 2017)
	D210Y [FIB]	CMT2A	
	V226_S229del	CMT2A	
	[FIB]/[MUS]	CMT2A	
	R274W [FIB]	CMT2A	
	M376V [FIB]/[MUS]	CMT2A	
	R707P [FIB]/[MUS]	CMT2A	
No change in mtDNA copy number	T105M [FIB]	CMT2A	Amiott et al. (2008)
	I213T [FIB]	CMT2A	
	V273G [FIB]	CMT2A	
	L734V [FIB]	CMT2A	
Increased mtDNA copy number	A383V [FIB]	CMT2A	(Sitarz et al., 2012; Rizzo et al., 2016)
	58 Variants [Total DNA from blood leucocyte samples]	CMT2A	
Decreased mitochondrial movement	V69F	CMT2A	(Baloh et al., 2007; Misko et al., 2010)
	L76P	CMT2A	
	R94Q	CMT2A	
	P251A	CMT2A	
	R280H	CMT2A	
	W740S [NEU]	CMT2A	
Increased lipid droplet abundance	R364W [FIB]	CMT2A	Larrea et al. (2019)
	M376V [FIB]		
	W740S [FIB]		
Decreased lipid droplet abundance with perinuclear distribution	D414V [FIB]	Sensorineural hearing loss and optic atrophy	Sharma et al. (2021b)
Increased mitophagy	A383V [FIB]	CMT2A	Rizzo et al. (2016)

### 2.1 MFN2 Variants and MERCs

Despite the controversy in knockout studies regarding the exact role of MFN2 in mediating MERCs, the existing data examining MERCs in the context of MFN2 CMT2A variants suggests that the pathogenic MFN2 variants studied to date all lead to reduced MERCs (Table 1). The first study to examine how MFN2 variants impact MERCs re-expressed the R94Q variant in MFN1/2 double knockout mouse embryonic fibroblasts (MEFs) and found that this variant was unable to restore the reduced abundance of MERCs (de Brito and Scorrano, 2008b). Similar re-expression strategies also failed to fully rescue MERCs with the P251A and R280H variants (Basso et al., 2018). In addition to a re-expression approach, MERCs have been examined in cells derived from CMT2A patients harbouring different MFN2 variants. One study looking at three different MFN2 variants (R364W, M376V and W740S), found an overall increased physical distancing between mitochondria and ER at MERCs in patient cells *via* EM (Larrea et al., 2019). However, the length of MERCs was increased for the R364W variant from a patient with a more severe clinical phenotype, possibly as a compensatory response. Further investigation into lipid metabolism and calcium homeostasis showed a variety of differences among the MFN2 variants, consistent with alterations to MERC functions. In a separate study of fibroblasts harbouring a homoplasmic D414V MFN2 variant from a patient showing atypical characteristics of CMT2A, a significant decrease in both the size and the number of MERCs was reported (Sharma et al., 2021b). Even though MFN2 is just one of many proteins involved in forming MERCs, the available data looking at pathogenic MFN2 variants all show altered MERCs (Larrea et al., 2019). These observations are consistent with the notion that MFN2 is an important mediator of MERCs (Naon et al., 2016), and with the idea that impaired MERCs may contribute to the disease pathology.

### 2.2 MFN2 Variants and Mitochondrial Fusion

Given the integral role of MFN2 in fusion, it is most often assumed that pathogenic MFN2 variants will have impaired fusion capabilities, leading to fragmented mitochondrial networks. However, as detailed below, this does not always appear to be the case (Table 1). With respect to mitochondrial morphology, perhaps the most characterized MFN2 variant is the R94Q variant, which is most often linked to fragmentation of the mitochondrial network in re-expression studies (Detmer and Chan, 2007; Misko et al., 2010; Wolf et al., 2019). There are certainly many other MFN2 variants that also appear to have deficient fusion function. For example, a study into motor neurons expressing MFN2 variants T105M, R274W, H361Y and H364W showed mitochondrial fragmentation (Franco et al., 2020). Meanwhile, mitochondrial fragmentation was also noted in fibroblasts from a patient with the MFN2 D414V variant, which is unusual due to its location a region of the HR1 domain that is largely devoid of MFN2 variants (Sharma et al., 2021b).

However, not all MFN2 variants investigated to date impact mitochondrial network morphology. For example, re-expression of V69F, L76P and R274Q in MFN1/2 double knockout MEFs can rescue mitochondrial morphology, indicating that these variants are fusion competent (Detmer and Chan, 2007). Conversely, several other variants (R94Q, R94W, T105M, P251A and R280H) were unable to rescue morphology. Meanwhile, expression of the L76P and W740S variants also led to aggregated mitochondrial clusters, a phenotype attributed to excessive mitochondrial tethering without subsequent fusion, which can also be induced by overexpressing wild-type MFN2 (Huang et al., 2007).

Further supporting the idea that not all MFN2 variants impact fusion, studies looking at patient fibroblasts harboring different variants observed normal mitochondrial network integrity, including the T105M, I213T, F240I, V273G or L734V variants (Amiott et al., 2008), as well as the R364W, M376V and W740S variants (Larrea et al., 2019). A possible explanation for the normal mitochondrial morphology in these patient cells, is the fact that fibroblasts also express MFN1, which may be able to compensate for the lack of MFN2 fusion function. Another explanation is that when fibroblasts are grown in media with glucose as an energy source they are not as dependent mitochondrial function for ATP, and thus they may not exhibit mitochondrial defects. With respect to this latter issue, fibroblasts containing the R274W MFN2 variant had fragmented mitochondrial networks when grown in galactose media, but not glucose media (Beresewicz et al., 2017). Thus, it may be worth examining mitochondrial morphology when fibroblasts are grown in galactose, rather than glucose. Especially for patient fibroblasts harbouring MFN2 variants where normal mitochondrial morphology has been reported previously.

Further complicating the picture of how pathogenic MFN2 variants impact mitochondrial fusion, several studies have shown hyperfused mitochondrial networks, which are not consistent with decreased fusion. For example, one study of fibroblasts from a patient carrying compound heterozygous missense G80V and R104Q variants in MFN2 showed an increase in the mitochondrial volume, and more connected mitochondrial networks (Codron et al., 2016). Meanwhile, introduction of the L76P and R364W mutations into the *Drosophila* homolog Marf, and expression in fly neurons also led to hyperfused mitochondrial networks (El Fissi et al., 2018). However, it should also be noted that hyperfused mitochondrial networks are not necessarily a result of increased fusion. To this end, a recent study showed that overexpressing the MFN2 R364W variant in multiple cell lines led to excessive mitochondrial hyperfusion compared to overexpression of wild-type MFN2, which was mediated by decreasing levels of DRP1, resulting in reduced fission (Das et al., 2021). In this regard, the hyperfusion linked to R364W overexpression may be an artefact and could also perhaps explain the excessive fusion described in *Drosophila* models. Nonetheless, with respect to how alterations in fusion may contribute to pathology, it is worth recalling that both excess fission and fusion are linked to peripheral neuropathy (Sharma et al., 2021a). Despite the caveats listed above, it seems safe to conclude that not all CMT MFN2 variants impact mitochondrial fusion to the same degree, nor in the same direction, if at all. Further supporting this notion, next we will highlight the variability in CMT2A variants with respect to two mitochondrial phenotypes that are affected by impaired fusion, mtDNA maintenance and respiration.

### 2.3 MFN2 Variants and mtDNA Maintenance

Loss of mitochondrial fusion can lead to both mtDNA depletion and mtDNA deletions, as well as impact the distribution of mtDNA throughout the mitochondrial network (Sabouny and Shutt, 2021), all of which have been reported for some MFN2 variants. Although one of the first studies in patient fibroblasts to examine mtDNA in the context of MFN2 variants (T105M, I213T, V273G and L734V) did not report changes to mtDNA copy number, nor the presence of deletions (Amiott et al., 2008), subsequent work noted the accumulation of mtDNA deletions in patient fibroblasts with the D210V variant, which was linked to an additional optic atrophy phenotype (Rouzier et al., 2012). Additionally, mtDNA deletions were reported in patient fibroblasts and in skeletal muscles of patients harbouring other MFN2 variants, including Q74R M376V, R707P and V226_S229del (Vielhaber et al., 2013). Other studies looking at tissue samples reported mtDNA depletion in skeletal muscle from a patient exhibiting early-onset CMT2A who harbored the D210Y MFN2 variant (Renaldo et al., 2012), while a two-fold decrease in the mtDNA copy number was observed in tissue samples from three patients carrying the MFN2 variants M376V, R707P or V226_S229 (Vielhaber et al., 2013). In contrast to the reports mentioned above, increased, mtDNA copy number was reported in leucocyte fractions from a group of 58 CMT2A patients (Sitarz et al. (2012), which could reflect cell-type differences. Finally, D414V fibroblasts have smaller mtDNA nucleoids, though the reason for this remains unknown (Sharma et al., 2021b).

Similar to fusion, the variable mtDNA phenotypes linked to certain MFN2 variants suggest that mtDNA impairments are not necessarily linked to the peripheral neuropathy phenotype in CMT2A patients. Instead, mtDNA impairments may be linked to other patient phenotypes associated with CMT2A. In this regard, MFN2 variants linked to mtDNA alterations are found in patients with optic atrophy, such as the D210V variant (Rouzier et al., 2012) and the D414V variant (Sharma et al., 2021b). Similarly, pathogenic variants in another mitochondrial fusion protein, OPA1, are also linked to optic atrophy (Amati-Bonneau et al., 2008; Hudson et al., 2008). Meanwhile, *in vivo* mouse studies show that eliminating mitochondrial fusion by knocking out both MFN1 and MFN2, or OPA1 leads to depletion of mtDNA copy number, as well as link elevated levels of mtDNA mutations to impaired mitochondrial fusion (Chen et al., 2010). These trends are consistent with the notion that reduced fusion may be responsible for mtDNA impairments. However, MFN2-mediated impairment of MERCs, which also play a role in marking sites of mtDNA synthesis (Lewis et al., 2016), could also contribute to mtDNA impairments in CMT2A.

### 2.4 MFN2 Variants and Altered Mitochondrial Respiration

Reduced mitochondrial respiration can be a downstream effect of several functions related to MFN2, including impaired mitochondrial fusion (Mourier et al., 2015), mtDNA depletion (Viscomi and Zeviani, 2017), or reduced MERCs (Rieusset, 2018). To this end, complete loss of MFN2 function can lead to reduced mitochondrial respiration, as shown in an MFN2 knockdown model (Yao et al., 2019), and in a conditional MFN2 mouse cardiac knockout model (Mourier et al., 2015). However, CMT2A MFN2 variants are not generally thought to be linked to impairment of mitochondrial respiration, as early studies using patient fibroblasts did not reveal differences for MFN2 variants, such as T105M, I213T and V273G (Amiott et al., 2008). Similarly, another study of patient fibroblasts harbouring the MFN2 variants R364W, M376V and W740S also showed no changes to mitochondrial respiration (Larrea et al., 2019). However, there are several exceptions showing that some MFN2 variants can impact mitochondrial respiration. For example, reduced maximal respiration was observed in fibroblasts from a patient harboring compound heterozygous M376V and V226_S229 MFN2 variants, which are also correlated with reduced mtDNA copy number (Vielhaber et al., 2013). Meanwhile, reduced respiration was observed in fibroblasts from patients carrying MFN2 M21V, R364Q and A166T (Loiseau et al., 2007), as well as D414V (Sharma et al., 2021b). Finally, re-expressing Mfn2 R94W in MFN1/2 double knockout MEFs also failed to rescue reduced oxygen consumption (Amiott et al., 2008; Strickland et al., 2014). Thus, MFN2 variants can impact mitochondrial respiration, though this does not always appear the case. In this regard, as noted above, MFN1 complementation and glucose growth media are caveats that may be able to mask MFN2 dysfunction in patient fibroblasts.

### 2.5 MFN2 Variants and Mitochondrial Motility

Among the 80+ genes implicated in CMT, many are known to regulate axonal transport of cellular components, including mitochondria, suggesting that impaired axonal transport can contribute to the pathology of peripheral neuropathy (Schiavon et al., 2021). In this sense, reduced mitochondrial transport in long peripheral neurons is proposed to lead to insufficient energy at the ends of axons. With respect to MFN2, a lack of defined mitochondrial motility was first noted in MFN1/2 double knockout MEFs (Chen et al., 2003). Some of the first studies to examine how pathogenic MFN2 variants impact mitochondrial motility observed decreased mitochondrial motility in distal axonal regions when patient variants (V69F, L76PM R94Q, P251A, R280H, H361Y and W740S) were expressed in rat dorsal root ganglion neurons (Baloh et al., 2007; Misko et al., 2010). More recently, a mouse model expressing the human T105M MFN2 variant showed reduced axonal transport of mitochondria (Franco et al., 2020). Excitingly, an intermittent treatment that restored mitochondrial fusion also rescued mitochondrial motility and improved neuromuscular degeneration in this mouse model (Franco et al., 2020).

While impaired mitochondrial transport seems to be common to all MFN2 variants explored to date, it should be noted that only a handful have been investigated. Moreover, the length of neurons and proper mitochondrial transport may not be the only factor involved in the pathology, especially considering some neurons in the human brain, which are unaffected by CMT, can be longer than mouse motor neurons that are (Schiavon et al., 2021). In this regard, mitochondrial quality control is another parameter that is proposed to contribute to CMT (Sharma et al., 2021a). However, while loss of MFN2 leads to an aberrant accumulation of unhealthy mitochondria (Chen et al., 2010; Song et al., 2017, 2015), to date there is little work looking at how CMT2A MFN2 variants impact mitophagy. Contrary to the knockout MFN2 studies, reduced mitochondrial content is reported in patient iPSC-derived motor neurons with the A383V MFN2 variant, likely due to enhanced mitophagy (Rizzo et al., 2016).

### 2.6 MFN2 Variants and Lipid Homeostasis

Interactions between mitochondria and lipid droplets are important, as mitochondria can either consume or produce lipids (Benador et al., 2018), depending on the metabolic needs of the cell (Boutant et al., 2017). The role of MFN2 in tethering mitochondria and lipid droplets has only recently been recognized, with MFN2 shown to interact with lipid droplets perilipin proteins PLIN1 (Boutant et al., 2017) and PLIN5 (Miner et al., 2022). However, there is not much known about how important this interaction is with respect to the pathology of CMT2A. Nonetheless, loss of MFN2 function in mouse adipose tissue shows an increase in number and size of intracellular lipid droplets (Boutant et al., 2017), while MEFs lacking MFN2 had larger lipid droplets when incubated with oleate to stimulate lipid droplet formation (McFie et al., 2016). Meanwhile in the context of disease, when homozygous, the R707W MFN2 variant is strongly tied to a lipodystrophy phenotype in patients (Sawyer et al., 2015; Masingue et al., 2017; Capel et al., 2018). To date, only a few studies have examined the effects of MFN2 variants with respect to lipid droplets. In fibroblasts from patients with either of the R364W, M376V or W740S variants, higher lipid droplet density was observed (Larrea et al., 2019). Conversely, reduced lipid droplet abundance and pronounced perinuclear distribution of lipid droplets was observed fibroblasts from a patient homozygous for the D414V variant (Sharma et al., 2021b). Although it is unclear exactly how MFN2 variants impact mitochondrial-lipid droplet contacts, and the potential functional consequences on mitochondrial function, there are several possibilities. Increased lipid droplet content could be due to reduced transport of lipids into mitochondria for consumption, or metabolic changes that favor lipid droplet biogenesis. Conversely, reduced lipid droplet abundance could be due to reduced lipid storage or increased lipid consumption. Meanwhile the observation of altered lipid droplet distribution could also be a result of impaired contacts between mitochondria and lipid droplets, or alterations to cellular metabolism. Despite this discrepancy in lipid droplet alterations in different patient fibroblast lines, which suggests that different MFN2 variants can impact lipid droplets in distinct ways, and the fact that only a few MFN2 variants have been studied, it is not unreasonable to suggest that lipid droplet impairments may contribute to the pathology of CMT2A, as impairment of lipid droplets is linked to motor neuron and neurodegenerative diseases (Pennetta and Welte, 2018; Farmer et al., 2020).

## 3 Discussion

Though initially discovered as a mitochondrial fusion protein, it is now well-established that MFN2 has multiple roles. Nonetheless, our mechanistic understanding of how MFN2 dysfunction leads to disease pathology is still incomplete. As reviewed above, only a handful of the 100+ pathogenic variants in MFN2 that cause CMT2A have been investigated functionally, and even then, most variants have not been characterized comprehensively. One of the key takeaway lessons from this review of the literature is that not all variants affect MFN2 functions in the same way. A key example of this variability is the fact that not all MFN2 variants appear to lead to reduced mitochondrial fusion. While this discrepancy could be in part due to not looking in the right cell type (e.g., fibroblasts rather than neurons), overexpression artifacts, or growth conditions, it seems clear that not all MFN2 variants impair fusion to the same degree. This conclusion is also supported by the variability in mtDNA maintenance and mitochondrial respiration, which can be downstream of mitochondrial fusion. Thus, despite the evidence that impaired fusion is linked to peripheral neuropathy (Sharma et al., 2021a), the evidence presented here suggests that impaired fusion may not be the primary cause of peripheral neuropathy in CMT2A.

Contrary to the variability seen with fusion, impaired MERCs and reduced mitochondrial motility seem to be consistent features observed with MFN2 variants, suggesting that these dysfunctions contribute to the peripheral neuropathy phenotype that is the hallmark of CMT2A. However, it is important to note that these MFN2 functions have not been investigated across MFN2 variants to the same extent as fusion. While impaired mitochondrial motility makes sense in the context of the axonal peripheral neuropathy of CMT2A, the exact reason for reduced mitochondrial transport in response to MFN2 dysfunction is unknown. One possible explanation could be due to impaired interactions between MFN2 and the transport proteins MIRO1/2. Another possibility, could be due to a ‘checkpoint’ that prevents the exit of dysfunctional mitochondria for transport along axons, as has been described with impairment of the mitochondrial fusion protein OPA1 (Zaninello et al., 2020).

Meanwhile, the fact that impaired MERCs are also implicated in several other neurological disorders (de Mario et al., 2017; Shirokova et al., 2020), is also consistent with the notion that dysfunctional MERCs are also likely to be relevant to the disease pathology of MFN2-linked CMT2A. In this context, Alzheimer’s disease (AD) was one of the first neurodegenerative diseases proposed to involve MERC dysfunction (Schon and Area-Gomez, 2010), and reduced length of MERCs and disruption of lipid transfer were reported in a rodent AD model (Adami et al., 2019). Furthermore, some proteins associated with AD are localized to MAMs (Area-Gomez and Schon, 2016; Pera et al., 2017; Area-Gomez et al., 2018). Parkinson disease (PD) is another neurodegenerative disorder known to involve impairments in MERCs (Basso et al., 2018; Moore and Holzbaur, 2018; Puri et al., 2019), one example being the fact that MIRO1 is a PD associated gene, with pathogenic variants showing impairments in MERCs, along with reduced mitochondrial motility and mitophagy (Berenguer-Escuder et al., 2020). Finally, amyotrophic lateral sclerosis (ALS) is also seen in conjunction with MERC dysfunctions (Puri et al., 2019), where impairment of a key MERC tethering proteins, VAPB (vesicle-associated membrane protein-associated protein B), leads to ALS (Chen et al., 2021). Overall, neurodegenerative diseases are a common theme linked to impaired MERCs.

With respect to how MERCs dysfunction may cause peripheral neuropathy, it is notable that MERCs can be linked to mitochondrial motility via their role in Ca^2+^ homeostasis, as Ca^2+^ levels can stimulate mitochondrial fission (Steffen and Koehler, 2018), and regulate mitochondrial motility (Wang and Schwarz, 2009; Zaninello et al., 2020). Thus, it is tempting to speculate that MERC disruptions could contribute to reduced axonal mitochondrial motility in CMT2A by impacting Ca^2+^. In this regard, impaired Ca^2+^ homeostasis and abnormal mitochondrial distribution has also been described in connection with loss of GDAP1, another mitochondrial outer membrane protein linked to CMT (Pla-Martín et al., 2013).

Another way that MERCs could impact mitochondrial motility is via mitochondrial fission (Lewis et al., 2016; Abrisch et al., 2020; Gao et al., 2021; Kleele et al., 2021; Sabouny and Shutt, 2021), which is required to generate mitochondrial fragments that can be transported. Whether or not the different types of fission events linked to either biogenesis or mitophagy (Kleele et al., 2021) are also involved in mitochondrial axonal transport remains unknown. It is tempting to speculate that ‘biogenesis’ fission, which may be coupled to mtDNA replication (Lewis et al., 2016), and is required for proper nucleoid distribution (Ilamathi et al., 2021), would precede anterograde transport to help deliver “fresh” healthy mitochondria to the cell periphery. With respect to retrograde mitochondrial transport, one early idea was that dysfunctional mitochondria need to be transported to the soma for mitophagy. However, we now know that mitophagy can also occur within axons, while fission events preceding retrograde transport do not necessarily lead to mitophagy (Ashrafi et al., 2014). Thus, MERCs could also be involved in fission events that precede transport, which would agree with the fact that both retrograde and anterograde mitochondrial transport are impaired in CMT2A (Mou et al., 2021).

With respect to some of the other functions performed by MFN2, such as lipid metabolism and mitophagy, we do not yet have a broad enough understanding of how they are impacted by different MFN2 variants to know whether they underlie the peripheral neuropathy seen in CMT2A. Moreover, as these functions are also influence by MERCs, it is difficult to tease apart how they are influenced by MFN2 and may impact pathology mechanistically.

Overall, our understanding of the mechanistic basis for how MFN2 dysfunction leads to disease phenotypes is complicated by the many functions performed by the protein. Importantly, not all pathogenic MFN2 variants affect MFN2 functions in the same way and do not have the same consequences on mitochondrial function. This fact, combined with the variability in CMT2A patient phenotypes, suggests that impairment of some MFN2 functions may lead to some of the additional patient phenotypes. For example, impaired fusion and mtDNA deletions seem to be linked with optic atrophy (Yu-Wai-Man et al., 2010). Whether impairment of other MFN2 functions can also be linked to specific disease pathologies remains to be determined (e.g., lipid droplets and lipomatosis). Meanwhile, impairment of MERCs and mitochondrial transport seem to be common features associated with MFN2 variants. While it is difficult to separate the many functions of MERCs and MFN2, as there is considerable overlap, we suggest that MERC dysfunction plays a significant role in the peripheral neuropathy pathology caused by CMT2A MFN2 variants.
